# Targeted fluorescent imaging of a novel FITC-labeled PSMA ligand in prostate cancer

**DOI:** 10.1007/s00726-021-03102-8

**Published:** 2021-11-20

**Authors:** Haoxi Zhou, Yu Gao, Yachao Liu, Yitian Wu, Yan Fang, Baojun Wang, Baixuan Xu

**Affiliations:** 1 Chinese PLA General Hospital, Chinese PLA Medical School, Beijing, 100853 China; 2 Department of Nuclear Medicine, Chinese PLA General Hospital, Beijing, 100853 China; 3 Department of Urology, Chinese PLA General Hospital, Beijing, 100853 China

**Keywords:** PSMA, FITC, Fluorescence imaging, Radical prostatectomy

## Abstract

In this study, we synthesized a novel fluorescein isothiocyanate (FITC)-labeled prostate-specific membrane antigen (PSMA) ligand (PSMA-FITC) via the Fmoc solid-phase synthesis method, and the application value of PSMA-FITC in targeted fluorescence imaging of PSMA-positive prostate cancer was evaluated. The PSMA ligand developed based on the Glu-urea-Lys structure was linked to FITC by aminocaproic acid (Ahx) to obtain PSMA-FITC. The new probe was evaluated in vitro and in vivo. Fluorescence microscopy examination of PSMA-FITC in PSMA(+) LNCaP cells, PSMA(−) PC3 cells, and blocked LNCaP cells showed that the binding of PSMA-FITC with PSMA was target-specific. For in vivo optical imaging, PSMA-FITC exhibited rapid 22Rv1 tumor targeting within 30 min of injection, and the highest tumor-background ratio (TBR) was observed 60 min after injection. The TBR was 3.45 ± 0.31 in the nonblocking group and 0.44 ± 0.13 in the blocking group, which was consistent with the in vitro results. PSMA-FITC is a promising probe and has important reference value for the development of PSMA fluorescent probes. In the future, it can be applied to obtain accurate tumor images for radical prostatectomy.

## Introduction

In recent years, optical imaging technology has been widely used in basic biomedical research, clinical diagnosis and treatment in various fields. In the field of basic science, fluorescent labeling technology, combined with a variety of microscopic imaging techniques, can be used in neurology, oncology, and pharmacy at the cellular and subcellular levels. Fluorescence imaging technology, combined with fluorescently labeled target molecule technology, can be used for specifically detecting certain tumors, tissues, cells, or even molecules. Therefore, biocompatible organic small-molecule fluorescent dyes have been rapidly developed and gradually used in clinical diagnosis and treatment (Han et al. 2017). For example, the combination of ICG with an optical imager can provide fluorescence imaging navigation during surgery to guide the removal of primary and metastatic tumor lesions (Shah et al. 2019). Among fluorescent dyes, FITC, an organic small-molecule fluorescent dye, has a maximum absorption wavelength of 490–495 nm and a maximum emission wavelength of 520–530 nm, emitting bright yellow-green fluorescence. The human eye is most sensitive to yellow-green. As a result, FITC has become one of the most commonly used fluorescein dyes in biomedical research (Hu et al. 2020).

PSMA, also known as glutamate carboxypeptidase, is expressed at low levels on the surface of normal prostate glands and hyperplastic prostate cells but is significantly upregulated in prostate cancer cells. It is, therefore, a specific molecular marker for prostate cancer. Radionuclide-labeled PSMA ligand small-molecule imaging agents have shown great clinical application value in the detection and treatment of prostate cancer (Kopka et al. 2017). The targeting of the PSMA ligand is performed through the pharmacodynamic group Glu-urea-Lys (Eder et al. 2008; Schwenck et al. 2017; Muller et al. 2019; Nurhan Ergül et al. 2018). We speculated that Glu-urea-Lys might be an excellent optical imaging probe material. In this paper, the Fmoc solid-phase synthesis method was used to label Glu-urea-Lys with FITC, which was subsequently used to synthesize PSMA-FITC. In vitro and in vivo studies were conducted to explore the function and use of PSMA-FITC as a targeting probe.

## Materials and methods

### General materials

All chemicals (reagent grade) were obtained from commercial suppliers and were used without further purification. FITC was purchased from Maclin Biochemical Technology Co., Ltd. (Shanghai, China). PIPE, NHS, OtBu, DIEA, and other reagents for probe synthesis were purchased from China Peptides Co., Ltd. (Suzhou, Jiangsu, China).

### Synthesis of PSMA-FITC

(1) One g of Fmoc-Lys(Dde)-Wang resin was used as the insoluble solid-phase carrier. Swell with 30 ml DCM for 1 h. (2) Thirty milliliters (30% PIPE in DMF solution) was added to the resin and allowed to react for 20 min to remove the Fmoc-protected moiety of the amino group. (3) After washing six times alternating between with 30 ml of DMF and DCM (repeat this step before each step), 8 eq DSC and 16 eq DIEA were added to the resin and allowed to react with the exposed amino group for 90 min to obtain the NHS structure intermediate. (4) 8 eq of H-Glu(OtBu)_2_ with an unprotected amino group and 16 eq of DIEA were added to the resin to react with NHS for 24 h to form a stable peptide bond structure. (5) 30 ml of 3% hydrazine in DMF solution was used to remove the Dde-protected group in the Lys side chain amino group to expose the amino group. (6) 8 eq Fmoc-Ahx-OH, 8 eq HOBT, and 8 eq DIC were added to the resin and allowed to react for 2 h to obtain a tripeptide. (7) 30 ml of 30% PIPE in DMF solution was added to the resin and allowed to react for 20 min to remove the Fmoc protection from Ahx and expose the amino group. (8) 8 eq FITC was added to the resin and allowed to react with 50% Py in DMF solution to obtain the final fluorescently labeled tripeptide with side chain protection. (9) 50 ml of TFA was used to remove the side chain OtBu protection to obtain a crude product. (10) After purification by HPLC, the final pure product was obtained.

### HPLC purity identification and MS analysis

The purity of PSMA-FITC was determined by reverse HPLC. The UV absorption spectra were detected at 220 nm, and a C18 reversed-phase column (5 μm, 250 × 4.6 mm) was used. The flow rate was 1 mL/min with the mobile phase starting with 70% solvent A (0.05% TFA + 2% acetonitrile) and 30% solvent B (0.05% TFA + 90% acetonitrile) and then was changed to 54% solvent A and 46% solvent at 16 min. The product was analyzed by mass spectrometry, and its molecular weight was determined.

### The binding affinity of PSMA-FITC and PSMA

The temperature of the incubator was set to 37 °C. Recombinant human PSMA protein was diluted to 0.4 mg/ml with HEPES buffer before use. NAAG was diluted to 160 mM in HEPES buffer before use. The PSMA-FITC probe was diluted in HEPES buffer to different concentrations: 400 mM, 40 mM, 4 mM, 400 nM, 40 nM, 4 nM, 400 pM, and 4 pM. Then, 25 μl of NAAG solution, 25 μl of probe solution, and 50 μl of PSMA recombinant protein solution were added to an EP tube and centrifuged onto a plate. After the solution reached the bottom, it was incubated at 37 °C for 1 h. Three parallel reactions were set for each group. The mixture was transferred to a metal bath set at 95 °C until the denaturation of the protein was terminated. Then, 100 μl of OPA detection reagent was added to each tube, mixed well, and protected from light. One hundred milliliters of the mixed solution was removed from each tube and transferred to a 96-well blackboard, and the microplate reader was immediately used for detection (Ex/Em = 350/450 nm, gain 100).

### Cell lines and culture conditions

The LNCaP, 22Rv1 and PC3 human prostate cancer cell lines were purchased from GuYan Biotech Co., Ltd. (Shanghai, China). The LNCaP cells, 22Rv1 cells and PC3 cells were cultured in RPMI-1640 medium (Gibco Life Technologies, Grand Island, NY, USA) supplemented with 10% fetal bovine serum (FBS) and 1% P/S in an incubator containing 5% CO_2_ at 37 °C.

### In vitro fluorescence imaging of PSMA-FITC

Both the LNCaP cells and PC3 cells were grown in a T25 cell culture flask (Corning Inc., Corning, NY, USA). The cultured LNCaP cells and PC3 cells were inoculated into a confocal dish and placed in an incubator at 37 °C/5% CO_2_ for 24 h. The medium was changed 2 h before the cell uptake experiment. The medium was removed, the flask rinsed once with PBS, and 5 μg of PSMA-FITC (100 μl) was added to each dish, which was then filled with medium to a final volume of 2 ml. The cells were incubated in an incubator for 60 min, washed 4 times with PBS buffer containing 0.2% BSA. Add 450 μl 4% paraformaldehyde to fix the cells for 10 min then rinse with PBS for four times. Cell nucleus were stained with 450 μl Hoechst33342 for 10 min and rinsed with PBS for four times. Add 500 μl PBS before observation. The cells were observed under confocal microscope (Olympus FV 1000) in dark room. In the blocking group, the LNCaP cells were pretreated with 2-PMPA (20 μg) for 30 min, and then PSMA-FITC was added and incubated for 60 min. The steps are the same as described above in the dark.

### Animal models

All animal procedures were performed in accordance with the protocol approved by the Animal Care and Use Committee of the PLA General Hospital. BALB/c male nude mice (approximately 3–4 weeks old, with a body weight of 13–15 g) were purchased from Charles River Laboratories (Beijing, China). Then, 0.1 ml (approximately 5 × 10^6^ cells) of 22Rv1 cell suspension was subcutaneously injected into the right shoulder of each mouse to form an 22Rv1 tumor xenograft. Vernier calipers were used to measure the length and shortest diameter of the tumor, and the tumor volume was estimated according to the formula (*V* = 1/2 × long diameter × shortest diameter^2^). Fluorescence imaging was performed in vivo and in vitro when the tumor volume reached 200–300 mm^3^. The PC3 cell planting method is the same as 22Rv1 cell planting method.

### In vivo and ex vivo fluorescence imaging

In vivo fluorescence imaging was performed using the French Biospace Lab in vivo optical imaging system, the PHOTON IMAGER OPTIMA multidimensional real-time awake animal in vivo optical imaging system software was used for analysis, and the FITC exclusive filter was used to collect the fluorescence of PSMA-FITC. All images were collected using the same parameters (lamp voltage, filter, exposure height, exposure time, and field of view). The collected images were processed in a unified manner, and the number of photons per second per centimeter squared per steradian (ph/s/cm^2^/sr) was used for analysis. 22Rv1 tumor-bearing mice in the nonblocking group (*n* = 5) and PC3 tumor-bearing mice (*n* = 5) were injected with PSMA-FITC (0.5 mg/kg) through the tail vein, and fluorescence imaging was performed at different time points after injection. The 22Rv1 tumor-bearing mice (*n* = 5) in the blocking group were injected with a mixture of 2-PMPA (2 mg/kg) and PSMA-FITC (0.5 mg/kg), and fluorescence imaging was performed 60 min after the injection. Another 9 22Rv1 tumor-bearing mice were divided into three groups (3/group), PSMA-FITC (0.5 mg/kg) was injected into the tail vein, and the mice were euthanized 30, 60, or 120 min after injection. The tumors, tissues, and organs were dissected, and a fluorescence imaging system was used for in vitro imaging. The ROI was delineated, and the average fluorescence intensity reported.

### Data processing and statistical analysis

All measured values are expressed as the means ± standard deviation. Independent sample *t*-test was used for statistical analysis, and *P* < 0.05 was considered statistically significant. To clarify tumor contrast, the region of interest (ROI) was delineated, and the mean fluorescence intensity of tumor tissue (T) and background (B) was collected. TBR was used to represent tumor-background contrast. Normal muscle tissue was selected as the background to outline.

### Acute toxicity test

ICR male mice (approximately 3–4 weeks old, with a body weight of 12–16 g) were injected with PSMA-FITC (100 mg/kg) through the tail vein, and organs were collected 7 days later. The organs were sectioned and stained with HE. The control group was injected with the same volume of normal saline. The steps are the same as described above.

## Results

### Synthesis of PSMA-FITC

**Fig. 1:**
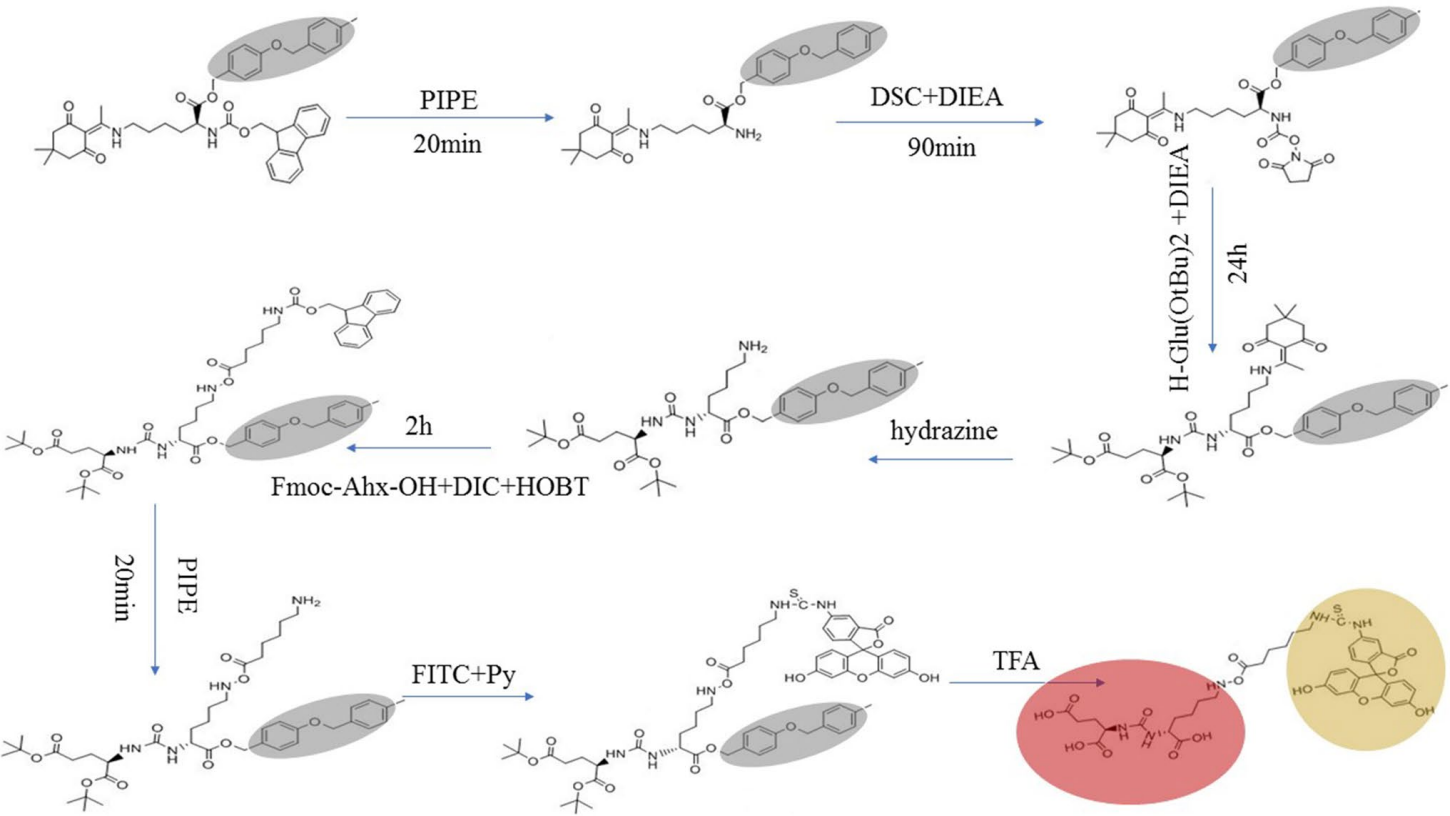
PSMA-FITC synthesis route map

The synthetic steps of PSMA-FITC are shown in Fig. 1.

### HPLC purity identification and MS analysis

**Fig. 2:**
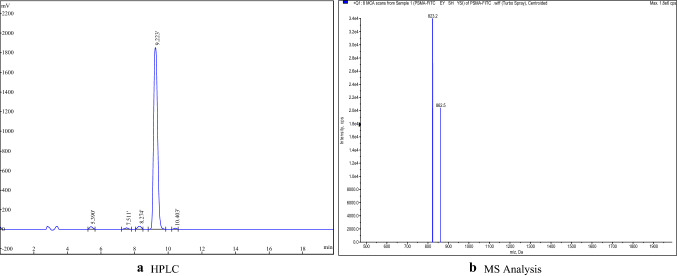
**a** HPLC, **b** MS analysis

HPLC purity analysis is shown in Fig. 2a; the purity of the synthesized product was 96.12%. The results of mass spectrometric analysis are shown in Fig. 2b; the molecular weight (M + H^+^) was 823.2.

### Binding specificity of PSMA-FITC

**Fig. 3:**
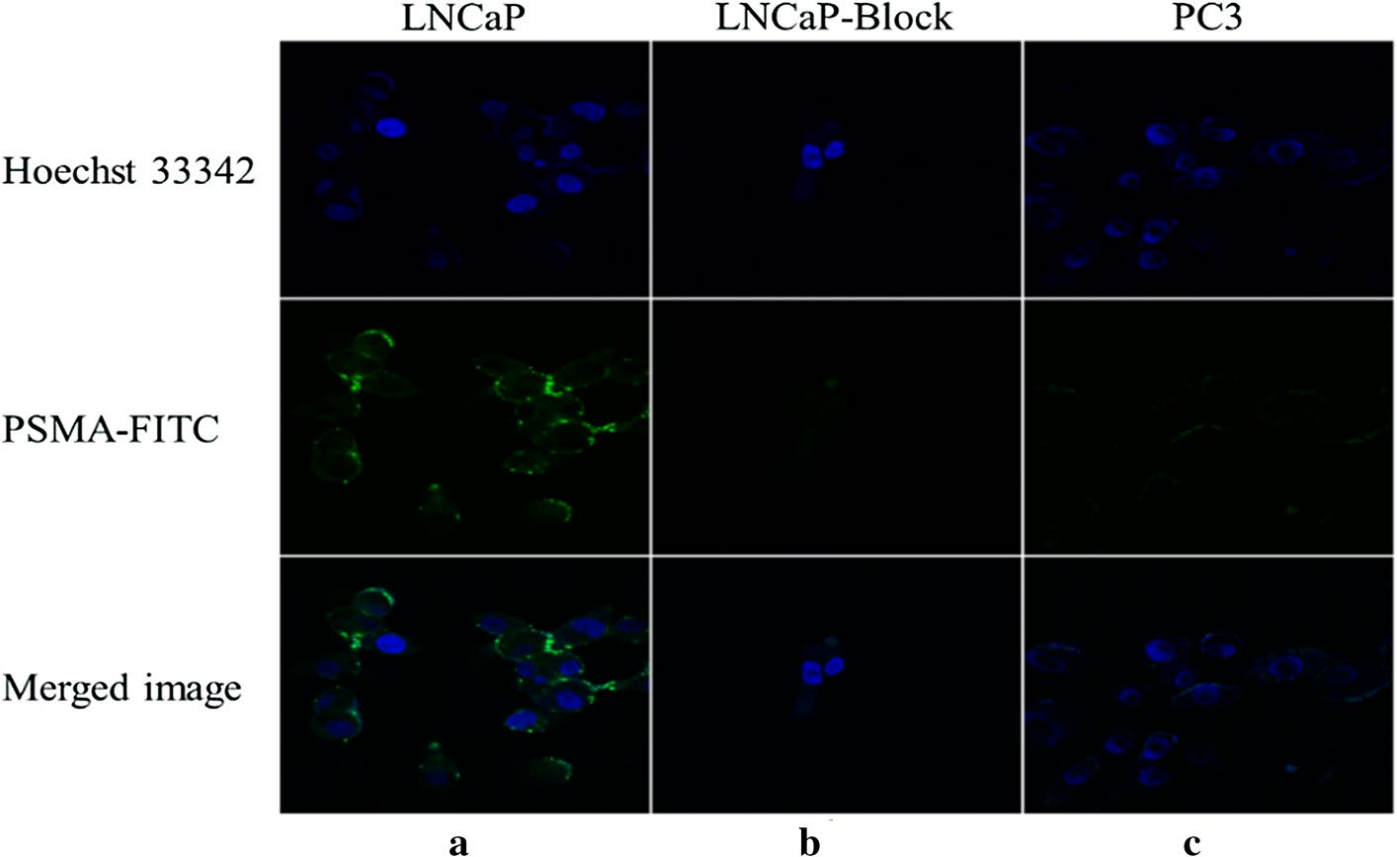
Fluorescence cell uptake experiments. **a** PSMA-FITC and LNCaP cells were incubated at 37 °C for 60 min. **b** After blocking with 2-PMPA for 30 min, the PSMA-FITC and LNCaP cells were incubated at 37 °C for 60 min. **c** PSMA-FITC and PC3 cells were incubated at 37 °C for 60 min

PSMA-FITC and LNCaP cells were incubated at 37 °C for 1 h, and then, confocal fluorescence microscopy imaging was performed. Significant fluorescence signals were seen on the membrane of LNCaP cells and in the cytoplasm, as shown in Fig. 3a. There was negligible fluorescence signaling in the 2-PMPA-blocked LNCaP cells (Fig. 3b) and PSMA (−) PC3 cells (Fig. 3c). In summary, confocal fluorescence microscopy results indicate that PSMA-FITC can specifically bind to PSMA.

### The binding affinity of PSMA-FITC and PSMA

The KI value of PSMA-FITC is 6.085E-010, which is nanomolar. The KI value of the PSMA blocker ZJ43 is 1.418E-009, and the affinity of PSMA-FITC to PSMA is higher than that of the PSMA blocker ZJ43.

### In vivo and ex vivo fluorescence imaging

**Fig. 4:**
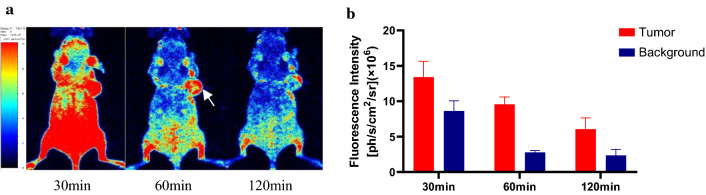
In vivo fluorescence imaging and ROI analysis. **a** Fluorescence imaging of 22Rv1 tumor-bearing mice after intravenous injection of PSMA-FITC (0.5 mg/kg). The tumor can be clearly seen from 30 to 120 min, and the fluorescence intensity is recorded as the number of photons per second per centimeter square per steradian (ph/s/cm^2^/sr). **b** Comparison and quantitative analysis of uptake of PSMA-FITC in tumor and background tissues. At each time point, the tumor uptake was significantly higher than the background uptake. Each error bar represents the standard deviation (*n* = 5)

**Fig. 5:**
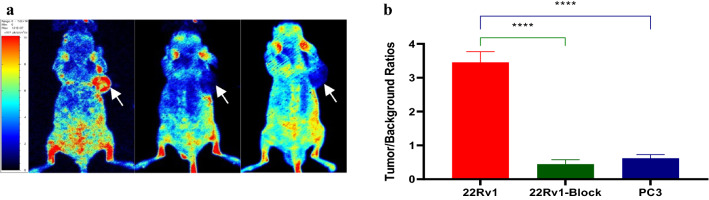
In vivo fluorescence imaging and TBR analysis. **a** Fluorescence imaging of the right shoulder of 22Rv1 tumor-bearing mouse 60 min after injection of PSMA-FITC (left). Coinjection of 2-PMPA blocked the tumor uptake of PSMA-FITC (middle). Fluorescence imaging of the right shoulder of PC3 tumor-bearing mouse 60 min after injection of PSMA-FITC (right). **b** In nonblocked or blocked 22Rv1 tumors and PSMA(-) PC3 tumors, the tumor-background fluorescence intensity ratio was based on the ROI analysis of PSMA-FITC uptake 60 min after injection (****P < 0.0001)

**Fig. 6:**
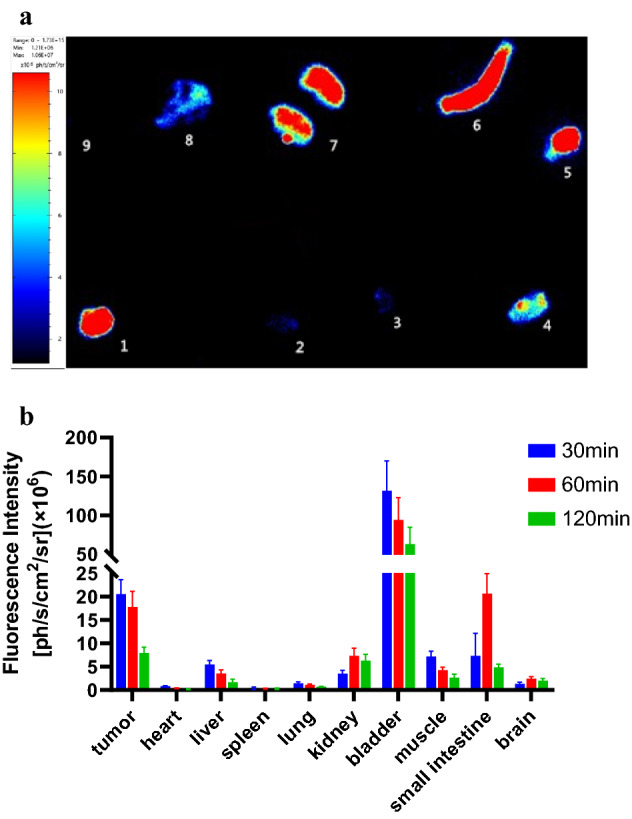
Distribution of PSMA-FITC in vivo. **a** Sixty minutes after injection of PSMA-FITC, the mice were euthanized, and the tumors, tissues and organs of the mice were dissected for in vitro imaging. 1: tumor, 2: heart, 3: lung, 4: muscle, 5: bladder, 6: small intestine, 7: kidney, 8: liver, 9: spleen. **b** ROI analysis of the fluorescence intensity of the main tissues 30, 60, and 120 min after injection of PSMA-FITC. Each error bar represents the standard deviation (*n* = 3)

Fluorescence images of 22Rv1 tumor-bearing mice were collected after the injection of PSMA-FITC (0.5 mg/kg) through the tail vein (Fig. 4a). Rapid tumor targeting of 22Rv1 was demonstrated 30 min after PSMA-FITC injection. The contrast between the tumor and the background was highest 60 min after injection. A histogram of the mean fluorescence intensity of the tumor and the background over time is plotted in Fig. 4b. The fluorescence intensity of the tumor and the background decreased with time. In contrast, normal tissue eluted the probe faster. In PC3 tumor-bearing mice with PSMA (−), the tumor uptake of the probe was significantly lower than that of 22Rv1 tumor-bearing mice. The specificity of PSMA-FITC against PSMA was verified by a blocking experiment. The mice in the blocking group were injected with a mixture of 2-PMPA (2 mg/kg) and PSMA-FITC (0.5 mg/kg). The results showed that a PSMA blocker significantly reduced the uptake of the probe by the tumor. Figure 5a shows fluorescence images of the 22Rv1 tumor-bearing mice in the nonblocking and blocking groups 60 min after injection. By outlining the ROI, the mean tumor fluorescence intensity was determined, the TBR was calculated 60 min after injection, and the TBR was found to decrease from 3.45 ± 0.31 to 0.44 ± 0.13 (*P* < 0.05) (Fig. 5b). In conclusion, PSMA-FITC can specifically bind PSMA, which is consistent with the results of in vitro cell experiments. In addition, in vitro imaging of the probe was performed to analyze the distribution of the probe in vivo. Nine 22Rv1 tumor-bearing mice were selected and divided into 3 groups (3/group). PSMA-FITC (0.5 mg/kg) was injected through the tail vein, and mice were euthanized at 30, 60, or 120 min after injection. Tumors, tissues, and organs were dissected for in vitro imaging (Fig. 6a, b).

### Acute toxicity test

**Fig. 7:**
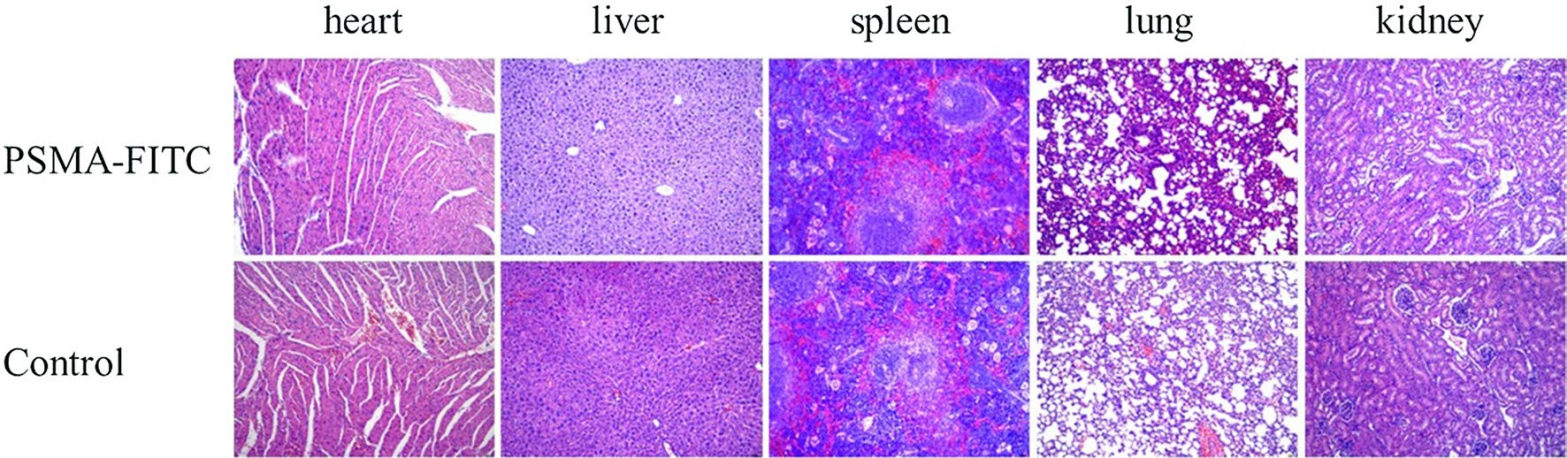
Histopathological analysis of mice by HE staining in acute toxicity test. The tissues included heart, liver, spleen, lung, kidney in PSMA-FITC (top) and control group (bottom)

After high-dose injection of PSMA-FITC, the mice organs were not damaged (Fig. 7). PSMA-FITC showed good safety.

## Discussion

Malignant tumors are the second leading cause of human death and seriously threaten human health (Siegel et al. 2019). Cancer precision medicine is advocated because it can be used to realize accurate, real-time dynamic tumor classification and diagnosis and to develop individualized tumor prevention and treatment plans (Letai 2017). Currently, the application of molecular imaging technology is not simply limited to the diagnosis of tumors, but is gradually being extended throughout the field of integrated diagnosis and treatment, showing good prospects. With the development of the discipline, important progress has been made in finding highly sensitive target molecules, constructing highly specific imaging probes, and establishing high-resolution imaging methods, all of which promote the rapid transformation of molecular imaging technology into clinical practice. Various imaging methods, including CT, MR, PET, SPECT, and optical imaging, have been widely used for the noninvasive assessment of anatomical abnormalities and changes in biological functions in vivo (Atreya et al. 2010). In terms of molecular imaging, PET has achieved great success in the clinical diagnosis of many tumors. For example, ^18^F-FDG is widely used in the imaging of highly metabolic tumors, and ^68^Ga-DOTA-TATE is a somatostatin receptor imaging agent that is widely used in the imaging of neuroendocrine tumors (Zhang et al. 2020). In the diagnosis of prostate cancer, PSMA-targeted PET imaging is an emerging method of prostate cancer imaging; its sensitivity and specificity are better than those of traditional imaging to a certain extent, and it greatly improves the detection rate of early prostate cancer (Chen et al. 2016). The PSMA ligand is also superior to other commonly used PET imaging agents (Pan et al. 2020; Rowe et al. 2016a, b). ^68^Ga-PSMA-11 was the first small-molecule PET imaging agent generated on the basis of Glu-urea-Lys, which has good biological distribution characteristics. ^18^F-DCFPyL is also a PSMA-specific small-molecule imaging agent developed based on the Glu-urea-Lys structure. It has the characteristics of high affinity and good pharmacokinetics in vivo. After clinical comparison, it was found that its performance is better than that of ^68^Ga-PSMA-11. It is currently the most commonly used PSMA-specific small-molecule probe in clinical practice.

Currently, molecular imaging technology is also used in the treatment of tumors, being used for intraoperative navigation for identification of tumors, evaluation of curative effects, drug delivery, immunotherapy, etc. Tian et al. used Cerenkov luminescence imaging (CLI) to evaluate the early response to chemotherapy in drug-resistant gastric cancer, and a high-sensitivity endoscopic CLI (ECLI) system was developed with a dual-mode deep cooling approach to improve the imaging sensitivity. Tumor resection on hepatocellular carcinoma-bearing mouse model was performed under ECLI guidance (Zhang et al. 2019; Liu et al. 2018). During surgery, determining whether the tumor borders and lymph nodes are involved is a challenge. Molecular imaging technology can provide precision for guiding tumor resection through intraoperative imaging. Currently, molecular imaging technologies that can be applied to intraoperative navigation include optics and MRI, especially optical molecular imaging technologies that have led to clinical transformation in tumor operations such as breast cancer, liver cancer, and glioma surgeries. For example, the fluorescent tracking dye ICG is used to detect breast sentinel lymph nodes in real time and dynamically during surgery (Chi et al. 2013). In radical prostatectomy, positive surgical margin (PSM) is one of the most common problems. To avoid urinary incontinence, the surgeon may separate too close to the apex of the prostate or close to the posterolateral prostate to avoid erectile dysfunction, which may increase the risk of PSM. However, PSM is a high-risk factor for postoperative biochemical recurrence. Patients with PSM need to receive adjuvant radiotherapy or combined adjuvant endocrine therapy after surgery, which can damage the neurovascular bundles (NVBs) preserved during surgery and directly affect the surgical effect. Nowadays, ICG can only be used to locate NVBs (Mangano et al. 2018). Therefore, it is of great significance to develop a new prostate cancer specific probe for navigational surgery. The tumor site and boundary will be determined intraoperatively, and the surgical resection scope can be clarified to reduce the incidence of PSM and retain NVB extremely. Takahito et al. used the combination of PSMA antibody J591 and ICG to achieve targeted fluorescence imaging of prostate cancer, but it takes 2 days for J591-ICG to be fully absorbed, internalized, and activated by the tumor (Nakajima et al. 2011). Therefore, developing fast-acting targeted molecular probes for use in vivo is important, as this type of probe will make it possible to accurately locate tumors during surgery. Based on the Glu-urea-Lys structure of ^18^F-DCFPyL, we replaced the nuclide-binding group in ^18^F-DCFPyL with FITC. To fully expose the Glu-urea-Lys group, Ahx was used to connect the two compounds together, which appropriately extended the distance between the fluorescent group FITC and the PSMA ligand and enabled the synthesis of PSMA-FITC (Fig. 1).

To test the tumor-targeting effect of PSMA-FITC, the affinity of the probe to PSMA was determined in vitro. The results show that the affinity is at the nanomolar level and that the affinity is better than that of the PSMA blocker ZJ43, which may be due to the benzene ring structure of the fluorescent group increasing the affinity for the PSMA ligand. In addition, we used 22Rv1 tumor-bearing mice for evaluation. In vivo optical imaging studies (Figs. 4, 5) showed that the PSMA-FITC nonblocking group had rapid 22Rv1 tumor targeting and a good TBR 60 min after injection (Fig. 4). The elution rate of the probe in the tumor was much slower than that in the normal tissue, and the contrast between the tumor and the normal tissue was good 60–120 min after injection. PSMA-FITC and 2-PMPA were coinjected for the blocking experiment. Sixty minutes after injection, compared with the nonblocking group, the uptake of 22Rv1 in the blocked group was significantly reduced (*P* < 0.05) (Fig. 5), indicating that PSMA-FITC is a target-specific probe. We then analyzed the dynamics of drug metabolism by in vitro optical imaging. The uptake of PSMA-FITC by the kidney is higher than that measured in other normal organs, and the fluorescence intensity of the bladder is obviously at a high level, which indicates that the kidney may be the excretion pathway of PSMA-FITC (Fig. 6). The metabolism of ^18^F-DCFPyL is similar (Wondergem et al. 2019). In addition, the liver and small intestine also showed strong fluorescence intensity, indicating that PSMA-FITC also has liver and intestine metabolism. Considering the results shown in this report, we plan to develop nuclear and fluorescent dual-modal probes with rapid targeting of prostate cancer in the future and develop molecular probes that integrate inspection, diagnosis, and intraoperative navigation. This work may provide an opportunity to reduce the incidence of positive margins and preserve neurovascular bundles during radical prostatectomy.

## Conclusion

A novel FITC-labeled PSMA ligand (PSMA-FITC) was synthesized by Fmoc solid-phase synthesis. PSMA-FITC has high sensitivity and specificity for PSMA(+) prostate cancer. The rapid targeting and high tumor-background contrast of PSMA-FITC proved that PSMA ligand-mediated prostate cancer-targeted fluorescence imaging has high potential value. This study provides a reference for the future development of PSMA nuclides and fluorescent dual-modal probes.

## Abbreviations

PSMA: Prostate-specific membrane antigen
FITC: Fluorescein isothiocyanate
Glu: Glutamate
Lys: Lysine
Ahx: Aminocaproic acid
ICG: Indocyanine green
PIPE: Piperidine
NHS: *N*-Hydroxysuccinimide
OtBu: Oxytert-butyl
DIEA: Diisopropylethylamine
DCM: Dichloromethane
DMF: *N*,*N*-Dimethylformamide
HOBT: 1-Hydroxybenzotriazole
DIC: *N*,*N*′-Diisopropylcarbodiimide
Py: Pyridine
TFA: Trifluoroacetic acid
HPLC: High-performance liquid chromatography
NAAG: *N*-Acetyl-aspartyl-glutamate
HEPES: N-2-Hydroxyethylpiperazine-N-ethane-sulfonic acid
OPA: *O*-Phthaldialdehyde
PBS: Phosphate-buffered saline
ROI: Region-of-interest
PK: Pharmacokinetics
PET: Positron emission tomography
CT: Computed tomography
^18^F-FDG: ^18^F-Fluorodeoxyglucose
